# The Molecular Basis of Polyunsaturated Fatty Acid Interactions with the *Shaker* Voltage-Gated Potassium Channel

**DOI:** 10.1371/journal.pcbi.1004704

**Published:** 2016-01-11

**Authors:** Samira Yazdi, Matthias Stein, Fredrik Elinder, Magnus Andersson, Erik Lindahl

**Affiliations:** 1 Max Planck Institute for Dynamics of Complex Technical Systems, Molecular Simulations and Design Group, Magdeburg, Germany; 2 Department of Clinical and Experimental Medicine, Linköping University, Linköping, Sweden; 3 Science for Life Laboratory, Stockholm and Uppsala, Stockholm, Sweden; 4 Theoretical and Computational Biophysics, Department of Theoretical Physics, KTH Royal Institute of Technology, Stockholm, Sweden; 5 Department of Biochemistry and Biophysics, Center for Biomembrane Research, Stockholm University, Stockholm, Sweden; University of Illinois, UNITED STATES

## Abstract

Voltage-gated potassium (K_V_) channels are membrane proteins that respond to changes in membrane potential by enabling K^+^ ion flux across the membrane. Polyunsaturated fatty acids (PUFAs) induce channel opening by modulating the voltage-sensitivity, which can provide effective treatment against refractory epilepsy by means of a ketogenic diet. While PUFAs have been reported to influence the gating mechanism by electrostatic interactions to the voltage-sensor domain (VSD), the exact PUFA-protein interactions are still elusive. In this study, we report on the interactions between the *Shaker* K_V_ channel in open and closed states and a PUFA-enriched lipid bilayer using microsecond molecular dynamics simulations. We determined a putative PUFA binding site in the open state of the channel located at the protein-lipid interface in the vicinity of the extracellular halves of the S3 and S4 helices of the VSD. In particular, the lipophilic PUFA tail covered a wide range of non-specific hydrophobic interactions in the hydrophobic central core of the protein-lipid interface, while the carboxylic head group displayed more specific interactions to polar/charged residues at the extracellular regions of the S3 and S4 helices, encompassing the S3-S4 linker. Moreover, by studying the interactions between saturated fatty acids (SFA) and the *Shaker* K_V_ channel, our study confirmed an increased conformational flexibility in the polyunsaturated carbon tails compared to saturated carbon chains, which may explain the specificity of PUFA action on channel proteins.

## Author Summary

Polyunsaturated fatty acids (PUFAs) are natural components of the lipid bilayer in e.g. heart and nerve cells. Despite occurring at low concentrations, PUFA effects on membrane proteins can be quite dramatic. For instance, PUFA molecules have been shown to modulate voltage-gated K^+^ channels and are used in dietary treatments of epilepsy. The effects of PUFA-channel interactions have been postulated to involve opening of K^+^ channels, but the molecular mechanism remains unknown. Our molecular dynamics simulations of the *Shaker* K^+^ channel embedded into a PUFA-enriched membrane propose specific molecular PUFA-channel interactions. A PUFA binding site was determined that enabled specific interactions of both the negatively charged PUFA head groups and the lipophilic carbon tails. The proposed location of a PUFA binding site suggest amino acid residue targets for experimental mutational analyses and can potentially be valuable to the design of antiepileptic drugs targeting ion channels.

## Introduction

Voltage-gated ion channels are tetrameric membrane-embedded proteins that open and close their central ion-conducting pore in response to changes in the voltage across the membrane. These channels rank as the third largest class of signal transduction proteins, following G protein-coupled receptors and protein kinases [1]. Controlling the propagation of nerve impulses, muscle contraction, and hormone secretion are a few of the critical physiological functions carried out by voltage-gated ion channels [2], which makes them attractive drug targets. The channels’ sensitivity to voltage can be modulated by a wide variety of molecules, such as toxins [3] and lipids [4]. Polyunsaturated fatty acids (PUFAs) are essential parts of cell membrane phospholipids of heart cells and neurons [5] and have been found to shift the voltage dependence of the *Shaker* voltage-gated K^+^ (K_V_) channel to enable channel activation via a proposed electrostatic mechanism [6, 7]. While the fatty acid modulatory effects have been extensively studied, in particular on voltage-gated ion channels [8], the molecular mechanism by which PUFAs modulate channel functioning remains an open question.

Each of the four homologous subunits of voltage-gated channels is composed of six transmembrane (TM) helices (S1-S6), with the first four (S1-S4) making up the voltage-sensor domain (VSD), and the last two (S5-S6) constituting the central ion-conducting pore. The gating charges are situated on helix S4 and respond to changes in voltage across the membrane by inducing movements of the helix relative to the remainder of the protein [9–11]. Several crystal structures describe K_V_ channels trapped in the activated state (with S4 in an "up" conformation) [12–14], while modeling and experimental approaches have reached agreement on a common deactivated state (with S4 in a "down" conformation) [15–18]. The S4 movements are conveyed to the S5-S6 pore via the S4-S5 linker to drive the opening and closing of the channel [19, 20]. Because burying the positive S4 residues in a hydrophobic environment would involve extensive energetic penalty, the gating charges pair up with negatively charged partner residues on helices S1-S3 [14] or lipid phosphate groups [21].

PUFA molecules are embedded within the lipid bilayer, and the modulation of K_V_ channel activity can in principle occur via indirect or direct effects, or a combination of the two. For example, addition of PUFAs to cardiac myocytes blocked sodium currents in Na^+^ channels by increasing the membrane fluidity, which is suggestive of an indirect effect [22]. On the other hand, direct PUFA interactions have been observed for several voltage-gated ion channels [23–28] and are often inferred by exclusion of indirect lipid bilayer effects. In this way, direct PUFA-mediated inhibition have been observed in K_V_1.5 channels [28], Na_V_ channels [23, 25], and Ca_V_ channels [24] and both activation and inactivation on the K_V_1.5 and K_V_2.1 channels [26]. In addition, mutational analyses pinpointed direct effects of PUFAs to inhibit Na_V_ channels targeting the S6 helix in the pore [27] and to activate K_V_ channels via the extracellular part of the VSD [29].

Because K_V_ channels are integrated components of the nervous system, their malfunction is often connected to disease. As a result, refractory epilepsy [30–32], which is triggered by K_V_1-type or K_V_7-type channel malfunction [33–38], can effectively be treated by ketogenic diets containing a high PUFA content [6, 32]. Even though ketogenic diets have been prescribed to patients since the 1920s [39], the underlying mechanisms by which it operates and prevents the epileptic seizures remain elusive. However, given the wide scope of PUFA effects on a range of voltage-gated ion channels, the mechanism of ketogenic diets likely include PUFA-channel interactions.

**Fig 1:**
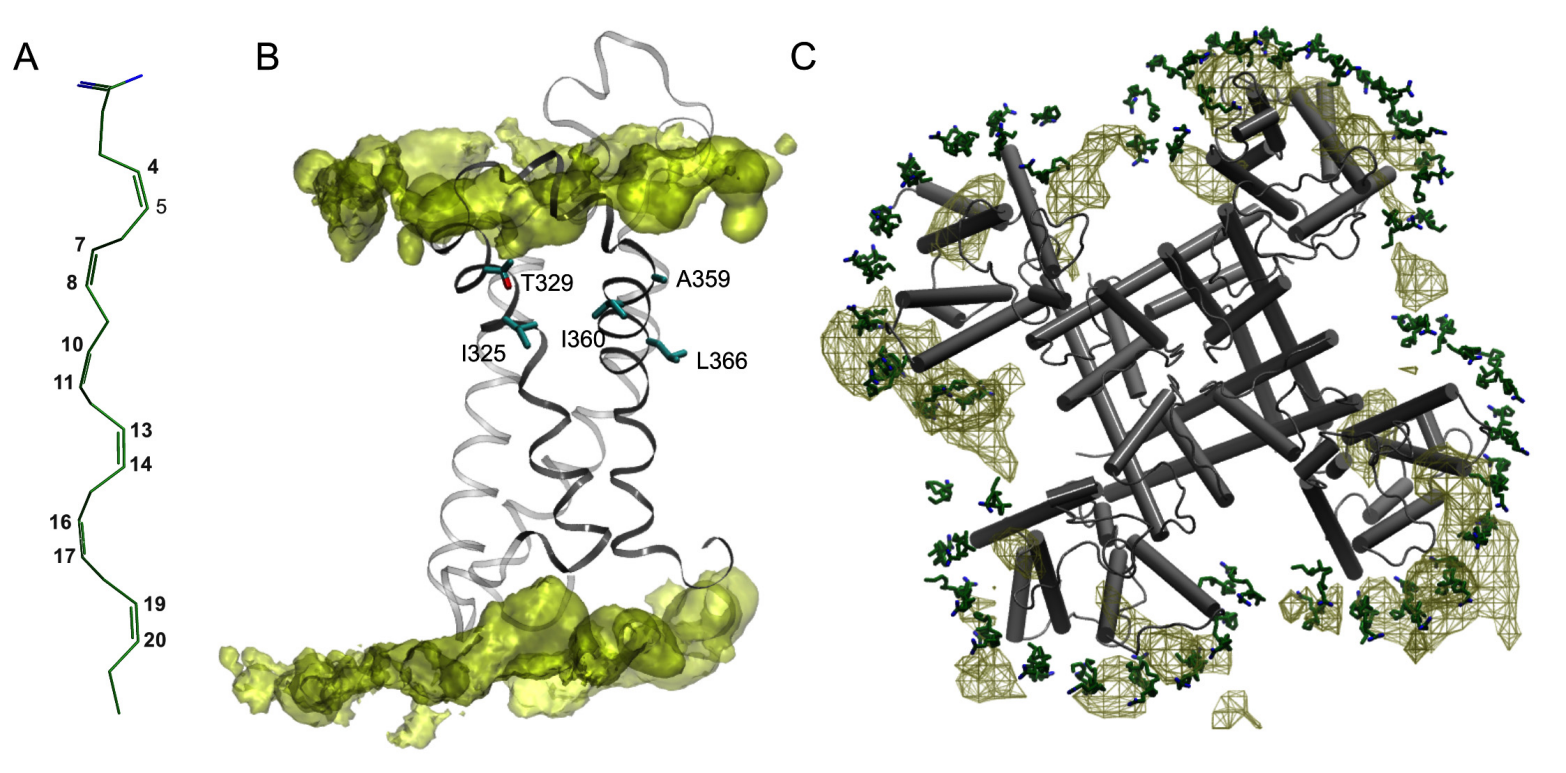
Molecular PUFA-VSD interactions in the *Shaker* channel. **(A)** Close-up of a PUFA (DHA) in its initial conformation. The numbers marked in grey depict the carbons forming the *cis* double-bonds. **(B)** Side-view of one VSD equilibrated in a POPC lipid bilayer represented by a yellow iso-density surface corresponding to the positions of lipid nitrogens in the simulation at 5% occupancy. The residues shown in experimental studies to be close to the interaction site of DHA, namely residues I325, T329 located on S3, and A359, and I360 located on S4, are colored in cyan [29]. **(C)** Top-view of the *Shaker* tetramer with PUFAs in their starting positions. The PUFA carboxyl head group and carbon tail are colored in blue and green, respectively. The simulated dynamics of the PUFAs surrounding the *Shaker* tetramer is represented by a brown mesh iso-density surface at 27% occupancy. The cut-off was chosen to visualize the differences between the PD and VSD interactions with the PUFAs.

The chemical signature of the PUFA molecule is a negatively charged carboxyl head group attached to a lipophilic acyl tail with two or more double bonds (Fig 1A). The PUFA head group was observed to activate *Shaker* K_V_ channels, which is referred to as the lipoelectric mechanism [7, 40] and can be abolished or reversed by neutralization of the negative PUFA head group charge or introducing a positive charge [7]. Similarly, the acyl chain properties have also proven important. While the acyl tail length does not seem to be critical, the number and geometry of the double bonds have significant effect on the modulatory properties of the PUFA [7], e.g. a minimum of two double bonds, particularly in the *cis* arrangement, substantially increase channel currents. PUFAs have been reported to modulate the gating mechanism of the *Shaker* K_V_ channel by partitioning into the lipid bilayer and interacting with the extracellular halves of the VSD helices S3 and S4 [29]. Specifically, a series of cysteine point mutations on the S3, S4, S5, and S6 helices revealed that residues on helices S3-S4 altered the sensitivity to the docosahexaenoic acid (DHA) PUFA. In addition, introducing positively charged cysteine-specific MTSEA^+^ probes identified four high-impact residues on the lipid-facing side of the VSD cytoplasmic region; I325 and T329 (helix S3) and A359 and I360 (helix S4) (Fig 1B). Indeed, channel VSDs make significant contacts with the surrounding lipid bilayer [20, 41, 42] and molecular dynamics (MD) simulations show specific interactions between the gating-charge R1 and R2 arginines on helix S4 on K_V_1.2 channels to salt-bridge to lipid head groups [43]. In addition, S4 gating charges have been observed to interact with lipid head groups in open, resting and intermediate states of the K_V_1.2 and the paddle–K_V_1.2 chimera channels [16, 17, 44]. Finally, the S3b-S4 paddle in VSDs of K_V_ channels has been identified as the key interaction point between the lipids and the channel [45]. Hence, a picture emerges where charged PUFAs partitioned into the lipid bilayer exert modulatory effects by contributing specific interactions with the VSD domains of ion channels.

In this work, we characterized PUFA-K_V_ channel interactions in the open and closed states of the channel using atomistic MD simulations. We explored the interaction between the *Shaker* K_V_ channel in an open state and a PUFA-enriched lipid bilayer and specifically characterized PUFA enrichment regions on the VSD. The open state *Shaker* K_V_ channel was modeled based on the high-resolution experimental structure of the chimera channel K_V_2.1/K_V_1.2 [14]. We found a potential PUFA-K_V_ channel site of interaction located on the lipid-facing side of a pocket connecting the extracellular halves of S3 and S4 helices, which is supported from experiments. In general, the lipophilic PUFA tail covered a wide range of non-specific hydrophobic interactions along helices S3 and S4, while the carboxylic head group formed fewer and more specific electrostatic interactions with the top regions of the S3-S4 helices and the S3-S4 linker. In addition, by performing simulations of saturated fatty acids (SFA)-K_V_ channel systems, the prerequisite of a polyunsaturated carbon tail is explained by simulations as suppression of flexibility in a saturated carbon tail. Our closed state simulations revealed an interaction pattern in which both the PUFAs and SFAs formed fewer interactions compared to the corresponding open state simulations. Together, our results explain the selective stabilization of the open state of a K_V_ channel, identify a putative PUFA interaction site at atomic detail and thereby provide novel K_V_ channel interaction points that can be tested experimentally and aid in the design of pharmaceutical compounds for the treatment of epilepsy.

## Results

In a set of independent simulations, we explored dynamics of polyunsaturated (docosahexaenoic acid, DHA) (Fig 1A) and saturated (docosanoic acid, DA) fatty acids in neat lipid bilayers as well as bilayers containing the *Shaker* K_V_ channel in both its open and closed states.

### Saturation levels in the carbon tail affect the structural dynamics

**Fig 2:**
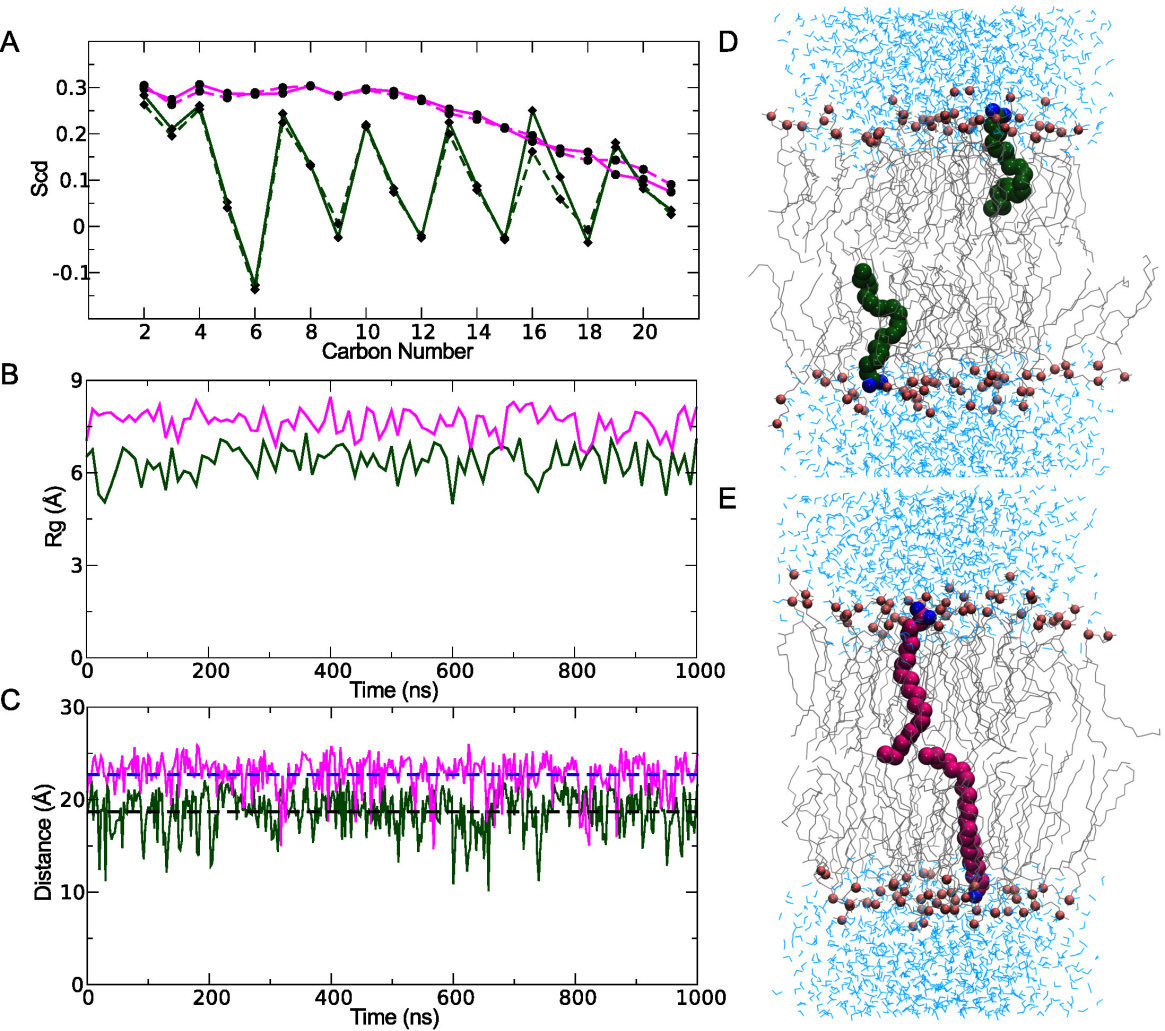
Structural flexibility in lipid-partitioned PUFA and SFA. **(A)** Deuterium order parameters of PUFA (green) and SFA (purple) carbon chains. **(B)** The radius of gyration of PUFAs (green) and SFAs (purple). **(C)** Distances between the head group oxygen and the last carbon in the PUFA (green) and SFA (purple) chains. The dotted lines denote the overall average distance for the PUFAs (black) and the SFAs (blue), respectively. Representative structures from 1 μs simulations of PUFAs (D) and SFAs (E) are shown embedded in POPC bilayers.

To determine how saturation levels in the fatty acid tail influenced the general structural dynamics, we simulated single PUFA (DHA) and SFA (DA) molecules in two separate 1-palmitoyl-2-oleoyl-sn-glycero-3-phosphocholine (POPC) membrane patches. As expected, the observed order parameters for both the PUFA and SFA molecules decreased gradually from the carboxyl end located in the bilayer interface to the end of the acyl chain tail in the bilayer center (Fig 2A). While the order parameters were quite similar near the carboxyl head and at the methyl end of both fatty acids, the PUFA order parameters were generally lower and displayed a different overall shape reflecting the positions of the *cis* double-bonds. Thus, the PUFA molecule exhibited greater conformational mobility compared to SFA in the POPC membrane patch.

Furthermore, we characterized differences in the overall shape and packing properties by measuring the radius of gyration of the carbon tail and head-to-tail length. The polyunsaturated chains displayed a significantly lower radius of gyration as compared to the fully saturated acyl chains (Fig 2B). Similarly, the distance between the carboxyl head group oxygen and the final methyl carbon in the tail (O-C_22_ distance) was 5 Å shorter in PUFAs compared to the SFAs (Fig 2C). In addition, the fluctuations in the O-C_22_ distance were more pronounced in the polyunsaturated carbon chains with extreme values between 10 Å and 20 Å. Hence, while the PUFA molecule tends to twirl and curl up (Fig 2D), the average SFA conformation is more extended (Fig 2E).

### PUFA interactions with the K_V_ *Shaker* channel in the open state

To characterize PUFA-protein interactions in the membrane, we initially distributed 32 PUFAs evenly across a POPC bilayer containing the *Shaker* tetramer, 16 on each bilayer leaflet (S1A Fig) and simulated for 5 μs (S1B Fig). Amino acids residues that resided within 3.5 Å of the PUFAs for more than 300 ns were mapped out in order to visualize the region of interaction between the channel and PUFAs on the outer leaflet of the membrane (S1 Table). The observed interactions differed significantly between the PUFA head and tail groups with the PUFA tails exhibiting approximately twice the number of contacts compared to the head groups. The contacting residues were distributed on the S1-S2 and S3-S4 segments of the VSD. Residues close to the PUFA head groups were either charged or polar and located on the outer regions of the VSD whereas residues contacting tail groups were predominantly hydrophobic and covered the inner parts of the S2 and S3 helices (S1C Fig). While this approach provided us with an initial idea of the PUFA interaction patterns, translational diffusion might prevent identification of realistic PUFA–channel interactions.

**Fig 3:**
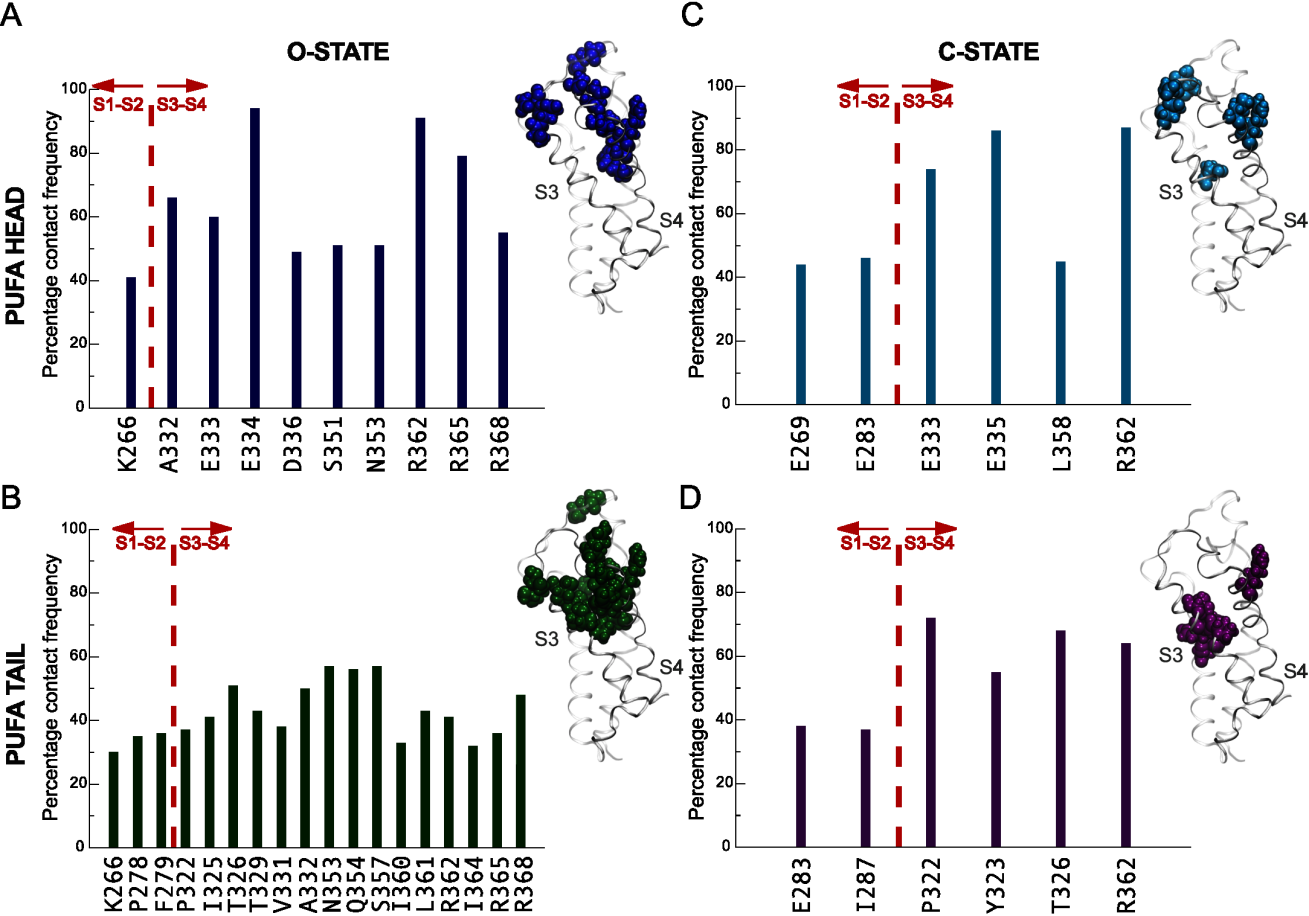
PUFA contacts to *Shaker* tetramer residues in the open and closed states. The contact frequencies of amino acid residues within 3.5 Å of PUFA carboxyl head groups and carbon tails are displayed for the open **(AB)** and closed **(CD)** states. The red dotted line differentiates contacts on helices S1 and S2 or helices S3 and S4 of the VSD. Side-view of a VSD and interacting residues are displayed separately for the PUFA carboxyl head groups and tails for each state of the channel (insets).

To increase sampling, we opted for a new system configuration starting with PUFAs packed around the VSD of each monomer in a semi-circular fashion (Fig 1C) using steered MD simulations. Starting from these initially closely packed positions, extensive sampling would in principle allow differentiation between specific and non-specific binding by monitoring PUFA structural dynamics given a PUFA diffusion constant of 4 x 10^−9^ cm^2^/s observed in the simulation. The resulting simulation trajectory showed the PUFAs to form clusters in the vicinity of helices S3 and S4 across all VSD subunits rather than pore helices S5-S6 (Fig 1C, green mesh surface). To identify interaction patterns, amino acid residues that resided within 3.5 Å of the PUFAs in the outer lipid leaflet for more than 300 ns were compared between the PUFA carboxyl head groups and carbon tails (Fig 3). All the reported contacting residues were positioned on the VSD and not on the pore domain. In addition, the interactions were significantly different between the PUFA head group and tail. While the PUFA carboxyl head groups engaged in fewer contacts but with higher contact frequencies (Fig 3A), the tails displayed a wider range of contacts occurring with lower frequencies (Fig 3B).

Protein residues contacting the PUFA tails were predominantly hydrophobic and distributed across the extracellular halves of the S1-S2 loop and helices S3 and S4 with 85% of the contacting residues localized on the extracellular halves of S3 and S4 (Fig 3B). The PUFA tail interactions to the protein displayed lower contact frequencies compared to the head groups, which indicates non-specific lipophilic interactions. In contrast, the majority of the contacts between the PUFA head groups and the protein were located on the top parts of the extracellular halves of S3 and S4, incorporating the S3-S4 linker and were either charged or polar residues displaying high contacting frequencies (Fig 3A). Specifically, the S3-S4 linker residues S351 and N353 formed hydrogen bonds with the PUFA head groups. In addition, the negatively charged carboxyl groups of residues E333, E334, and D336 located on the S3-S4 linker interacted with the negatively charged PUFA head groups mediated by Na^+^ ions. Presence of 100 mM NaCl neutralized the simulation system and sodium ions are typically observed in the vicinity of the protein (S2 Fig). An additional PUFA-protein electrostatic interaction with relatively high contact frequency is mediated by R362, which is one of the gating charges (R1). Parenthetically, we also observed interactions between the S4 gating charges R365 (R2), and R368 (R3) and one PUFA head group. These interactions were enabled by a nosediving movement of that particular PUFA molecule into the water-filled extracellular crevice. Because this PUFA-protein interaction appeared only once in our simulations, we will not ascribe significant physiological relevance to the interaction per se, but rather point out that PUFA interactions to the gating charges are structurally possible in a dynamic system.

### PUFA interactions with the K_V_ *Shaker* channel in the closed state

**Fig 4:**
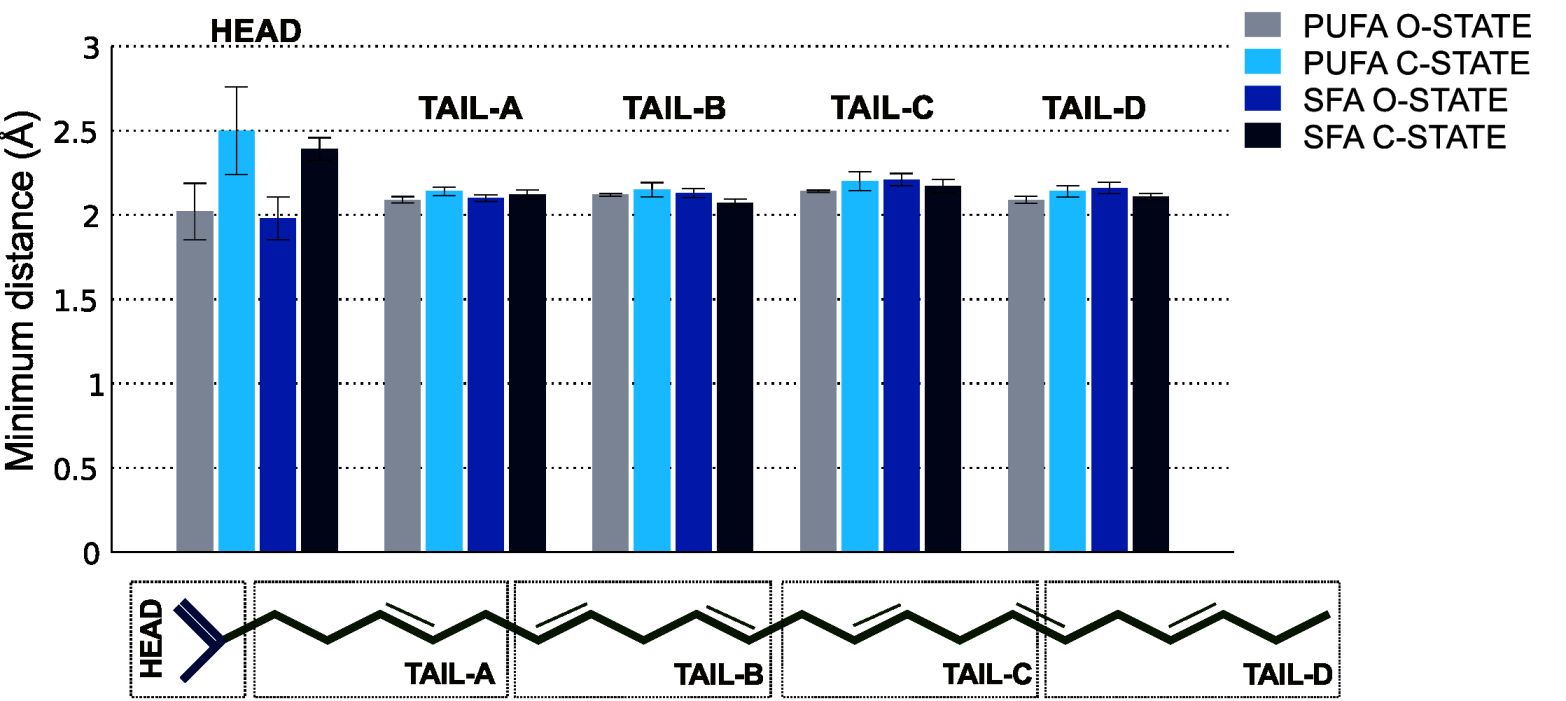
The average minimum distances between the channel in its open and closed states and the PUFAs and SFAs. The fatty acids were sectioned into five parts consisting of carbons C_2-6_ (TAIL-A), C_7-11_ (TAIL-B), C_12-16_ (TAIL-C), C_17-22_ (TAIL-D), and the head group (HEAD). The error bars indicate the standard error of the minimum distances across the four subunits of the channel.

To investigate the PUFA interactions with the K_V_ *Shaker* channel in the closed state, we generated a system consisting of a closed-state model with PUFA molecules in similar starting configurations as for the open-state system and simulated for 1 μs. The observed packing environment differed in-between PUFAs in the open and closed states of the channel as displayed by the results of contact analyses. In general, significantly fewer PUFA interactions were made in the closed-state simulation with an equal distribution of the number of contacts between the head and tail groups (Fig 3C and 3D). In addition, only half of these contacts were observed in the open-state simulation. To further differentiate the PUFA interaction pattern between the open and closed states of the channel, we measured minimum distances between different segments of the fatty acids and the channel (Fig 4). The major differences in minimum distances to the channel were found in the head groups with PUFA carboxyl head groups being 0.5±0.2 Å closer in the open state. In contrast, no significant variations between open and closed states were observed in the minimum distances between the channel and different sections of the PUFA tails.

### SFA interactions with the K_V_ *Shaker* channel in the open and closed state

**Fig 5:**
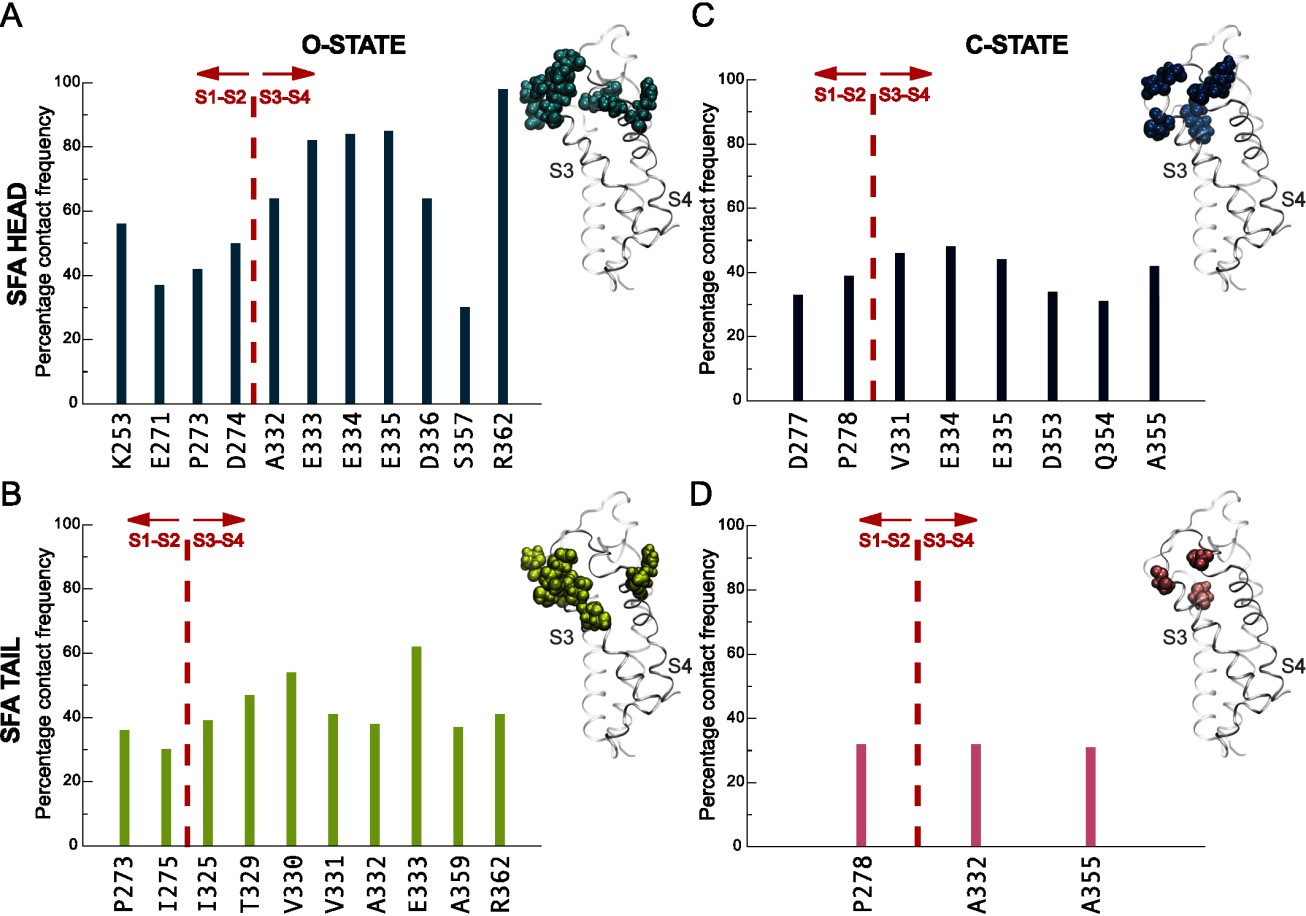
SFA contacts to the *Shaker* tetramer in the open and closed states. The contact frequencies of amino acid residues within 3.5 Å of SFA carboxyl head groups and carbon tails are displayed for the open **(AB)** and closed **(CD)** states of the channel. The red dotted line differentiates between helices S1 and S2 or helices S3 and S4 of the VSD. Side-view of a VSD and interacting residues are displayed separately for the PUFA carboxyl head groups and tails for each state of the channel (insets).

Because SFAs do not exert the modulatory effects of PUFAs, we explored SFA interactions with the channel in the open and closed states. The minimum SFA-channel distances showed a pattern that was identical to that observed for the PUFAs (Fig 4). The SFA carboxyl head groups were on average 0.4±0.1 Å closer to the channel in the open state, while no significant differences were detected across the different sections of the tail. However, the numbers and contact frequencies of interactions made by the SFA carboxyl head groups and carbon tails to the protein differed significantly between the open and closed states. SFA interactions for both head and tail regions in the closed state were lower both in number and contact frequency compared to the open state interactions (Fig 5).

Interestingly, the specific protein residues involved in PUFA and SFA interactions differed significantly. While almost all protein residues contacting PUFA head groups in the open state were located on helices S3-S4, the contacting residues were shifted to helices S1-S2 for the SFAs (Figs 3A and 5A) and the PUFA tail interactions in the open state were more numerous than for SFAs (Figs 3B and 5B). In addition, about half of the contacts between the protein and the SFA head and tail groups were completely unique. The specific contacts and their corresponding frequencies across the four subunits of the channel are reported in the S2 Table. Finally, the specific contact patterns seem independent of the overall dynamics since there were no significant differences in residency times between PUFAs and SFAs in neither open nor closed states (S3 Fig). However, while the distribution of the POPC residency times indicated more dynamics compared to PUFAs, this difference was reduced in the vicinity of four VSD residues shown experimentally to be involved in PUFA modulation of the *Shaker* channel [29]. In this position, three POPC molecules accompanied one single PUFA and reported 1 μs residency times.

### Electrostatic interactions characterize the PUFA interaction sites

**Fig 6:**
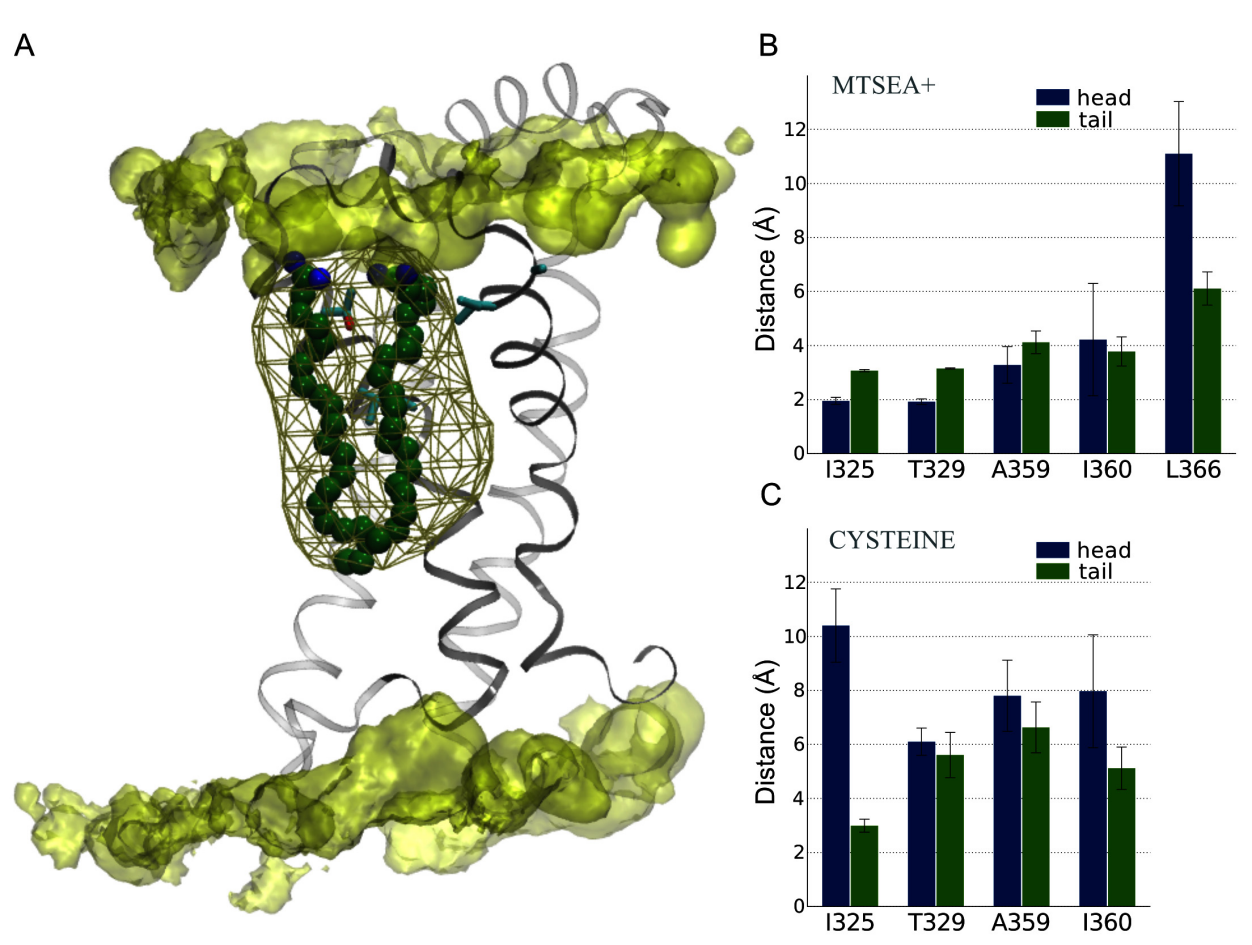
Characterization of PUFA interaction sites by introduction of MTSEA^+^ and cysteine probes. **(A)** Side-view of PUFAs interacting with the VSD and a PUFA iso-density surface at 14% occupancy depicted in brown mesh. **(B)** Average minimum distances between MTSEA^+^-modified residues and PUFA carboxyl head groups (blue) and carbon tails (green). **(C)** Average minimum distances between the cysteine-mutated residues and PUFA carboxyl head groups (blue) and PUFA carbon tails (green). The error bars display the standard errors of the VSD-PUFA minimum distances.

In the open state PUFA simulation, PUFAs were observed to cluster in the lipophilic pocket of the VSD at the extracellular end of the channel proximal to the S3-S4 linker with the hydrophobic tail tucked between helices S3 and S4 (Fig 6A). To characterize the identified PUFA interaction sites, we turned to reproduction of experimental data. Mutating residues I325 (S3), T329 (S3), A359 (S4), and I360 (S4) to cysteine and subsequent modification using positively charged MTSEA^+^ affect the PUFA-induced shift of the *Shaker* K_V_ channel [29]. Therefore, these four residues were proposed to directly modulate function by interactions to PUFA molecules. We introduced positively charged MTSEA^+^ cysteine labels at positions I325, T329, A359, and I360 in the open state and simulated for 1 μs. The minimum distances between the MTSEA^+^ mutated residues (I325, T329, A359, I360) and the PUFA head groups showed significant interactions varying between 2–4 Å (Fig 6B). To further verify this result, we included a negative control also originating from experimental data; mutating the L366 residue did not induce PUFA-mediated shifts in *Shaker* activation [29]. Indeed, the minimum distance between the PUFA head group and L366 remained larger than 10 Å during 500 ns of simulation, which is in stark contrast to the PUFA-sensitive mutations (Fig 6B).

In addition, to differentiate between the role of the PUFA head groups and tails and their specific interactions with the channel, we also monitored distances between the carbon tails and the mutated residues. In these analyses, the carbon tails were restricted to carbons C_12-22_ in the PUFA carbon chain near the terminal methyl group to enable better discrimination between PUFA head and tail. In contrast to PUFA head groups, the PUFA carbon tails maintained a ~1 Å larger minimum distance for all mutations except the I360 mutation which has a slightly lower average distance although a larger standard error in the head group minimum distance may account for this divergence (Fig 6B). A similar tendency was observed for the control mutation L366, where the PUFA tails displayed a significantly lower minimum distance to the mutated residue. In summary, the MTSEA^+^ simulation systems validated the modes of PUFA-protein interactions observed in the WT system where the PUFA head group interacted specifically to the S3-S4 linker while the tails maintained less defined protein interactions.

Considering the evident influence on the PUFA-channel interaction caused by MTSEA^+^-modified residues, we introduced an additional test to probe whether the effects remained after the removal of the added charge. At 500 ns, the MTSEA^+^ modified residues were mutated back into cysteines and were simulated for an additional 500 ns. Upon charge removal, a distinct change in the average distances between the cysteine side chains and the PUFA head groups was observed (Fig 6C), with PUFAs on average displaying a minimum distance of 6–10 Å across the four mutated systems. Similarly, the distances between the PUFA carbon tails and the cysteines were significantly altered, except for the I325 mutation (Fig 6C).

## Discussion

The general effects of PUFAs on both channel activation and inactivation has been documented for K_V_ channels [26, 28], Na_V_ channels [22, 27, 46, 47], and Ca_V_ channels [27, 48], but exactly where these unsaturated fatty acids bind remains less clear. It is also not ascertained whether it is the ion pore or the VSD that are the targets of action. For instance, reduction of K_V_1.1 channel currents were assigned to PUFA-protein interactions involving hydrophobic residues lining the cavity of the ion pore in the open state [49]. In contrast, a mutational study proposed a PUFA-interaction site to be located on the VSD, specifically the lipid-facing surface of the extracellular side of TM helices S3 and S4 [29].

Atomistic MD simulations are designed to monitor structural dynamics of complex environments, such as a membrane protein inserted into a lipid environment and solvated by water and ions. Therefore, the MD simulation approach is highly suitable for identifying and monitoring PUFA-protein interactions on the molecular level, which would be difficult to determine experimentally. Because a PUFA-binding site has never been characterized in atomistic detail, we set out to identify and characterize a potential PUFA binding site. The observed PUFA interaction site was located on the lipid-facing side of TM helices S3 and S4 on the extracellular side of the *Shaker* K_V_ channel in its open state, in agreement with previous experimental findings [29]. In addition, we observed a significant difference in the structural dynamics and protein interaction patterns of the PUFA carboxylic head group and the lipophilic carbon tail. The PUFA head groups favored fewer and highly specific polar/charged residue interactions along the S3-S4 extracellular part, in contrast to the carbon tails that interacted non-specifically with a wider range of S3-S4 residues buried in the hydrophobic core in the lipid bilayer. Furthermore, by monitoring the structural dynamics displayed by SFAs and PUFAs, we ascertained an increased conformational flexibility in the polyunsaturated carbon tails compared to saturated carbon chains. Because the saturation level of fatty acids are highly correlated to modulation of channel function [7, 47, 50, 51], the structural dynamics of fatty acids in the lipid bilayer are likely important to obtain optimal interactions to the channel protein. Our results indicate that the flexibility of the unsaturated carbon tail allows the fatty acid to visit several binding modes that enable a strong specific interaction between the carboxyl head group and the channel, which would otherwise be inaccessible to a more rigid saturated carbon tail. A similar conclusion was drawn from recent experimental studies of the *Shaker* channel [7]. To test this predicted interaction we performed simulations in the presence of SFA molecules. Indeed, the saturated carbon tails of the SFAs displayed significantly less interactions to the protein compared to the PUFA tails in the open state of the channel. In the closed state, both PUFAs and SFAs formed different interaction patterns compared to the open state simulations. A reduced number of interactions, in particular in the carbon tail interactions, contrasted the open and closed state interactions.

The increasing flexibility with unsaturation level of fatty acids is well documented. For instance, NMR studies have shown DHA to undergo rapid conformational transitions with short correlation times and exceptionally low deuterium order parameters [52, 53]. In addition, quantum mechanical calculations have shown that polyunsaturated chains sample greater conformational space around the *cis* double bonds with more rapid reorientations near the methyl end of the chain [54]. The increased conformational flexibility in PUFAs has been explained by lowered torsional energy barriers for the rotatable bonds in these carbon chains [55]. In addition, several rhodopsin studies show PUFA-specific modulation [55, 56]. Therefore, tail flexibility enabling PUFA electrostatic head group interactions may be a general mechanism.

The interactions between the PUFA head groups and the VSD in the open state were concentrated to a few residues displaying high contact frequencies. While all interactions did not occur across all four subunits, sampling interactions across subunits for extended microsecond simulations times enabled identification of the major interaction residues. Interestingly, one of these residues was R362, which is the first (R1) of the gating charge residues. Because involvement of gating charges has also been observed in *Shaker* K_V_ experiments [29, 57], it is indeed possible that PUFAs affect channel function by binding to our proposed binding site and reach the R1 gating charge from this position. In a recent study, mutations of residues M356 and A359 into arginines increased the K^+^ channel’s sensitivity to PUFAs considerably [57]. These residues, which are positioned on the S4 helix, also show up in our contact analyses but with lower contact frequencies, which might reflect limited sampling. In a study by Xu *et al*., it was established that higher hydrophobicity in a ten-residue segment in the extracellular part of the S3 helix in the paddle chimera helps to stabilize the open state of the channel [58]. Out of these ten residues, residues I325, T326, T329, V331, and A332 were identified as close contacts to PUFAs in our simulations of the open state channel, indicative of the role PUFAs play in stabilizing the open state of the channel.

Further comparison of our observed PUFA-protein interaction pattern to that of other lipid modulators such as the phosphatidylinositol-4,5-biphosphate (PIP_2_) lipids hints at significant complexity. Recently, PIP_2_ lipids were found to stabilize the closed state of K_V_7.1 channels by interacting with the lower residues of S4. However, in an open state PIP_2_ lipids migrated to the pore domain (PD) to form salt bridges with the S6 terminus and in this way meditated coupling between the VSD and the PD [59]. PIP_2_ lipids were also observed to migrate from the S4-S5 linker to the S2-S3 linker in KCNQ_2_ K_V_ channels to control deactivation kinetics [60]. Thus, MD simulations have identified putative structural features underlying the function of modulatory agents that appear fundamentally different. Future experimental efforts are needed to verify the interaction patterns presented in our study. Finally, our simulations studies provide a structural framework for future studies aimed at determining the free energy associated with K_V_ channel activation and deactivation in the presence of modulatory agents.

## Methods

### *Shaker* channel system setup in open state

The K_V_ *Shaker* channel has been extensively studied with respect to PUFA modulation (gating function) [6, 7, 29]. Because of a 66% sequence identity in transmembrane (TM) helices S0-S6 between the *Shaker* K^+^ channel and the high-resolution X-ray structure of the K_V_2.1 paddle–K_V_1.2 chimera channel (PDB ID 2R9R) [14], the structural model of the *Shaker* in an open state was based on this structure. A *Shaker* channel with partially truncated S3-S4 linker (residues 337–350) has been shown to have similar biophysical properties to the wild-type channel [61, 62]. Therefore, we opted for removing this extended loop, which is also missing in the chimera crystal structure. The three-dimensional structure of the *Shaker* channel was constructed with Modeller 9.12 [63]. Initially, five models were built and the optimal model was identified using a combination of the following model quality scores: The Discrete Optimized Protein Energy (DOPE) score, a statistical potential used to assess homology models [64], and the GA341 score, a method used for model assessment based on the percentage sequence identity between the template and the model [65].

The ion channel was immersed in a pure POPC bilayer [66] consisting of 256 lipids per leaflet using the g_membed tool in the GROMACS tool repository [67]. After inserting the protein into the bilayer 438 POPC lipids remained. The system was energy minimized with steepest descent until the maximum force reached < 1000 kJ/mol/nm and was subsequently equilibrated for 10 ns while keeping the protein and the waters position restrained (Fc = 1000 kJ/mol/nm^2^). With the position restraints removed, the system was relaxed in a 50 ns simulation. The Amber ff99SB-ILDN force field [68] was used for the construction of the membrane-protein system, in combination with the Berger force field [66] for the lipids and the TIP3P water model [69] and the CHARMM36 force field [70] was used for the 1 μs production run.

### PUFA and SFA system setup in open state simulations

Force field parameters for the PUFA (docosahexaenoic acid), SFAs (docosanoic acid) and the MTSEA^+^ compounds were obtained from the multipurpose atom-typer for CHARMM (MATCH) server [71], which utilizes libraries of topology and parameter files in existing force fields for extrapolation to the new molecules consistent with the parameterization strategy within a given force field. The default Charmm General Forcefield (top_all36_cgenff) was used for deriving partial charges and parameterization. The partial charges for the PUFA, SFA, and MTSEA^+^ molecules are presented in S3 Table. The calculated order parameters for DHA (see Fig 2A) are in agreement with those obtained previously both by computational and experimental efforts [54]. In addition, the order parameters for the SFA agree with the increased level of disorder towards the end of the chain reported for the saturated palmitic acid [54]. Together, these comparisons validate the parameterization procedure.

The simulations were performed using the all-atom CHARMM36 force field [70] and a development version of Gromacs [72, 73]. The LINCS algorithm [74] was applied for constraining bond lengths. Electrostatic interactions were calculated with the Particle-Mesh Ewald algorithm at every step [75]. P-LINCS [76], a non-iterative parallel constraints algorithm allowing replacement of hydrogens with virtual interaction sites, enabled 5 fs time steps [77]. A 1.2 nm cutoff was used both for electrostatics and van der Waals interactions, with neighborlists updated every 10 steps. The simulations were performed at constant pressure of 1.0 bar with Parrinello-Rahman pressure coupling [78] and the semiisotropic pressure scaling, time constant of 2.0 ps, and a system compressibility of 4.5e-5 bar^-1^. The temperature of the system was maintained at 300 K using the velocity-rescaling algorithm [79].

PUFAs were placed around each VSD on each lipid leaflet (Fig 1C) and clashing POPC lipids were removed resulting in 80 PUFAs and 275 POPC lipids surrounding the tetrameric *Shaker* channel. The system was subsequently solvated with 33,389 TIP3P waters and 192 sodium and 104 chloride ions replaced water molecules to neutralize the net system charge (and obtain a salt concentration of 0.1 M). The total number of atoms in the system reached 163,139. Initially, the system was equilibrated for 30 ns while keeping the protein, waters, and PUFAs frozen. Position restraints were then applied to the protein, PUFAs, and z-coordinates of the waters followed by 100 ns equilibration. Hereafter, all position restraints were removed except for the PUFAs and the system was allowed to equilibrate for an additional 10 ns. This series of equilibration steps allowed POPC lipids to pack around the PUFAs while preventing water molecules from penetrating into the membrane bilayer. The SFA-channel system was created by replacing the PUFAs in this equilibrated system with SFAs followed by a 200 ps equilibration step.

Two initial PUFA distribution patterns were tested. In the first, 32 PUFAs were evenly distributed across the lipid bilayer (S1A Fig) and sampling was performed in a 5 μs production run. In the second setup, we applied center of mass (COM) pulling using an umbrella potential between each VSD and the surrounding PUFAs in order to allow for close (but non-specific) protein-PUFA packing. Here, the PUFAs were pulled in the x and y dimensions with a harmonic force constant of 1000 kJ mol^-1^ nm^-2^ at a rate of 0.08–0.01 nm/ps depending on the initial distances between the PUFAs and the VSD followed by a 1 μs production run. The COM pulling procedure was repeated to create the SFA system.

### Modeling of *Shaker* channel in closed state

A model of the *Shaker* channel in a closed state was built based on a previous Rosetta model of the channel in the C3 state [18] with a partially truncated S3-S4 linker using Modeller 9.12 [63]. This closed-state model replaced the open state channel in the previously set up PUFA and SFA systems. The new system configurations were relaxed for 10 ns followed by COM pulling to position PUFA and SFA molecules in initial positions close to the channel and subsequent 1 μs production simulation runs.

To assess the structural integrity of the structures, the root mean square deviation (RMSD) of the protein backbone was calculated against the average structure for all four systems (PUFA and SFA channel systems in open and closed states) and compared to a channel-only system (S4 Fig). Overall, the channels maintain stable structures, settling around an RMSD of 1.5–2.0 Å throughout the simulation time.

### In silico mutagenesis of the VSD

Residues I325, T329, A359, I360, and L366 were mutated to cysteines and modified to include the reagent MTSEA^+^ to characterize PUFA interaction sites. The mutations were carried out using PyMOL (The PyMOL Molecular Graphics System, Version 1.3 Schrödinger, LLC). Each mutated system was minimized with a steepest descent algorithm until a maximum force of < 1000.0 kJ/mol was reached followed by a short 200 ps equilibration. Production runs were 1 μs long for each of the MTSEA^+^ mutated systems, except for the L366 system which was simulated for 500 ns. In addition, the MTSEA^+^ modified systems were mutated back to cysteines after 500 ns of simulations and continued for 500 ns.

### Small-scale PUFA and SFA systems

Two membrane patches were built using the membrane generator MemGen [80] with 24 lipids per leaflet. In each of the bilayer patches, DHA or DA, were inserted in each lipid leaflet and were set up as the described PUFA system. The systems each contained ~12,000 atoms with roughly 48 POPC lipids, 1,660 water molecules and 20 sodium and chloride counter ions. Each system was subjected to steepest descent energy minimization until the maximum force reached a value < 1000 kJ/mol/nm followed by 1.5 ns of equilibration. Production runs were 1 μs for each system configuration.

## Supporting Information

S1 Fig PUFA-*Shaker* interactions in a 5 μs MD simulation. (A) Top-view of the *Shaker* tetramer with 16 PUFAs on each leaflet of the membrane. The PUFA carboxyl head group and carbon tail are colored in blue and green, respectively. (B) Lateral displacement (x,y dimensions) of PUFAs in the membrane bilayer over 5 μs with the protein COM centered in the box. **(C)** Interacting VSD residues presented separately for the PUFA carboxyl head groups (blue) and carbon tails (brown).
(EPS)

S2 Fig Presence of Na^+^ ions. The number of sodium ions within 3.5 Å of the channel during the 1 μs simulations of the PUFA and SFA systems in the open and closed states and the channel-only system.
(EPS)

S3 Fig Number of PUFA/SFA/POPC molecules within 2 Å of the channel. Residence time for the number of PUFA, SFA (A) and POPC molecules (B) in the open and closed states of the channel. (C) Residence time for the number of PUFA and POPC molecules in the open state of the channel in vicinity of four VSD residues shown experimentally to be involved in mediating the PUFA effect.
(EPS)

S4 Fig Structural stability of the channel. Backbone RMSD of the channel relative to the average structure for the PUFA and SFA systems in the open and closed states of the channel and the channel-only system.
(EPS)

S1 Table PUFA interactions with the channel in 5 μs MD simulation. Amino acid residues within 3.5 Å of PUFA carboxyl head groups and carbon tails in the open state of the channel during the 5 μs simulation with a frequency longer than 300 ns.
(DOCX)

S2 Table PUFA/SFA interactions with the channel. Amino acid residues within 3.5 Å of PUFA and SFA carboxyl head groups and carbon tails in the open and closed states of the channel during the 1 μs simulation with a frequency longer than 300 ns.
(DOCX)

S3 Table Partial charges for PUFA, SFA, and MTSEA^+^. (DOCX)
