# Supplementary material for: The Molecular Basis of Polyunsaturated Fatty Acid Interactions with the Shaker Voltage-Gated Potassium Channel
Source: PLoS Comput Biol. 2016 Jan 11;12(1):e1004704. doi: 10.1371/journal.pcbi.1004704 (PMC4709198; doi:10.1371/journal.pcbi.1004704)
Supplement: S1 Table — Amino acid residues within 3.5 Å of PUFA carboxyl head groups and carbon tails in the open state of the channel during the 5 μs simulation with a frequency longer than 300 ns. (DOCX) [file pcbi.1004704.s005.docx]

| **PUFA O-STATE (5 μs)** | | | | | | | |
| --- | --- | --- | --- | --- | --- | --- | --- |
| **Head** | | | | **Tail** | | | |
| **Residue** | **% contact frequency** | **Occurrence in number of subunits** | **TM region** | **Residue** | **% contact frequency** | **Occurrence in number of subunits** | **TM region** |
| K266 | 13 | 1 | S1-S2 | V272 | 9 | 1 | S1-S2 |
| D270 | 14 | 1 | S1-S2 | T276 | 8 | 1 | S1-S2 |
| D274 | 27 | 1 | S1-S2 | D277 | 12 | 1 | S1-S2 |
| D277 | 13 | 1 | S1-S2 | F280 | 29 | 1 | S1-S2 |
| E333 | 8 | 1 | S3-S4 | L281 | 10 | 1 | S1-S2 |
| E335 | 29 | 2 | S3-S4 | T284 | 11 | 1 | S1-S2 |
| S352 | 15 | 1 | S3-S4 | I288 | 9 | 1 | S1-S2 |
| S357 | 19 | 1 | S3-S4 | Y323 | 12 | 1 | S3-S4 |
|  |  |  |  | F324 | 9 | 1 | S3-S4 |
|  |  |  |  | L327 | 16 | 1 | S3-S4 |
|  |  |  |  | T329 | 7 | 1 | S3-S4 |
|  |  |  |  | V330 | 13 | 2 | S3-S4 |
|  |  |  |  | V331 | 8 | 1 | S3-S4 |
|  |  |  |  | E333 | 7 | 1 | S3-S4 |
|  |  |  |  | S352 | 11 | 1 | S3-S4 |
|  |  |  |  | M356 | 8 | 1 | S3-S4 |
|  |  |  |  | S357 | 11 | 1 | S3-S4 |
