# Supplementary material for: The Molecular Basis of Polyunsaturated Fatty Acid Interactions with the Shaker Voltage-Gated Potassium Channel
Source: PLoS Comput Biol. 2016 Jan 11;12(1):e1004704. doi: 10.1371/journal.pcbi.1004704 (PMC4709198; doi:10.1371/journal.pcbi.1004704)
Supplement: S2 Table — Amino acid residues within 3.5 Å of PUFA and SFA carboxyl head groups and carbon tails in the open and closed states of the channel during the 1 μs simulation with a frequency longer than 300 ns. (DOCX) [file pcbi.1004704.s006.docx]

| **PUFA O-STATE** | | | | | | **PUFA C-STATE** | | | | | |
| --- | --- | --- | --- | --- | --- | --- | --- | --- | --- | --- | --- |
| **Head** | | | **Tail** | | | **Head** | | | **Tail** | | |
| **Residue** | **% contact frequency** | **Occurrence in number of subunits** | **Residue** | **% contact frequency** | **Occurrence in number of subunits** | **Residue** | **% contact frequency** | **Occurrence in number of subunits** | **Residue** | **% contact frequency** | **Occurrence in number of subunits** |
| K266 | 40 | 1 | K266 | 30 | 2 | E269 | 44 | 1 | E283 | 38 | 1 |
| A332 | 65 | 1 | P278 | 35 | 3 | E283 | 46 | 1 | I287 | 37 | 1 |
| E333 | 34 | 3 | F279 | 36 | 2 | E333 | 74 | 2 | P322 | 72 | 1 |
| E334 | 94 | 1 | P322 | 36 | 1 | E335 | 86 | 2 | Y323 | 55 | 1 |
| S336 | 48 | 1 | I325 | 41 | 2 | L358 | 45 | 1 | T326 | 68 | 1 |
| N353 | 51 | 2 | T326 | 50 | 2 | R362 | 87 | 1 | R362 | 64 | 1 |
| S357 | 51 | 2 | T329 | 42 | 2 |  |  |  |  |  |  |
| R362 | 90 | 1 | V331 | 37 | 1 |  |  |  |  |  |  |
| R365 | 78 | 1 | A332 | 49 | 3 |  |  |  |  |  |  |
| R368 | 55 | 1 | N353 | 57 | 1 |  |  |  |  |  |  |
|  |  |  | Q354 | 56 | 1 |  |  |  |  |  |  |
|  |  |  | S357 | 57 | 2 |  |  |  |  |  |  |
|  |  |  | I360 | 32 | 2 |  |  |  |  |  |  |
|  |  |  | L361 | 42 | 3 |  |  |  |  |  |  |
|  |  |  | R362 | 40 | 1 |  |  |  |  |  |  |
|  |  |  | I364 | 32 | 2 |  |  |  |  |  |  |
|  |  |  | R365 | 36 | 2 |  |  |  |  |  |  |
|  |  |  | R368 | 48 | 1 |  |  |  |  |  |  |

| **SFA O-STATE** | | | | | | **SFA C-STATE** | | | | | |
| --- | --- | --- | --- | --- | --- | --- | --- | --- | --- | --- | --- |
| **Head** | | | **Tail** | | | **Head** | | | **Tail** | | |
| **Residue** | **% contact frequency** | **Occurrence in number of subunits** | **Residue** | **% contact frequency** | **Occurrence in number of subunits** | **Residue** | **% contact frequency** | **Occurrence in number of subunits** | **Residue** | **% contact frequency** | **Occurrence in number of subunits** |
| K253 | 56 | 1 | P273 | 36 | 1 | D277 | 33 | 1 | P278 | 32 | 2 |
| E271 | 37 | 1 | I275 | 30 | 2 | P278 | 39 | 1 | A332 | 32 | 2 |
| P273 | 42 | 1 | I325 | 39 | 1 | V331 | 46 | 1 | A355 | 31 | 1 |
| D274 | 50 | 3 | T329 | 47 | 1 | E334 | 48 | 1 |  |  |  |
| A332 | 64 | 1 | V330 | 54 | 1 | E335 | 44 | 1 |  |  |  |
| E333 | 82 | 1 | V331 | 41 | 2 | N353 | 34 | 1 |  |  |  |
| E334 | 84 | 1 | A332 | 38 | 1 | Q354 | 31 | 1 |  |  |  |
| E335 | 85 | 1 | E333 | 62 | 1 | A355 | 42 | 1 |  |  |  |
| D336 | 64 | 1 | A359 | 37 | 1 |  |  |  |  |  |  |
| S357 | 30 | 2 | R362 | 41 | 1 |  |  |  |  |  |  |
| R362 | 98 | 1 |  |  |  |  |  |  |  |  |  |
