# Supplementary material for: The Molecular Basis of Polyunsaturated Fatty Acid Interactions with the Shaker Voltage-Gated Potassium Channel
Source: PLoS Comput Biol. 2016 Jan 11;12(1):e1004704. doi: 10.1371/journal.pcbi.1004704 (PMC4709198; doi:10.1371/journal.pcbi.1004704)
Supplement: S3 Table — (DOCX) [file pcbi.1004704.s007.docx]

| **PUFA** | | **SFA** | | **MTSEA+** | |
| --- | --- | --- | --- | --- | --- |
| **Atom** | **Charge** | **Atom** | **Charge** | Atom | Charge |
| O | -0.76 | C | 0.62 | N | -0.31 |
| C | 0.62 | C2 | -0.28 | CA | -0.09 |
| O3 | -0.76 | C3 | -0.18 | C | 0.51 |
| C4 | -0.28 | C4 | -0.18 | O | -0.51 |
| C5 | -0.18 | C5 | -0.18 | CB | -0.1 |
| C6 | -0.15 | C6 | -0.18 | SG | -0.08 |
| C7 | -0.15 | C7 | -0.18 | CE | -0.1 |
| C8 | -0.18 | C8 | -0.18 | NH | -0.3 |
| C9 | -0.15 | C9 | -0.18 | SD | -0.08 |
| C10 | -0.15 | C10 | -0.18 | CZ | 0.21 |
| C11 | -0.18 | C11 | -0.18 | H | 0.31 |
| C12 | -0.15 | C12 | -0.18 | HA | 0.09 |
| C13 | -0.15 | C13 | -0.18 | HB1 | 0.09 |
| C14 | -0.18 | C14 | -0.18 | HB2 | 0.09 |
| C15 | -0.15 | C15 | -0.18 | HE1 | 0.09 |
| C16 | -0.15 | C16 | -0.18 | HE2 | 0.09 |
| C17 | -0.18 | C17 | -0.18 | HZ1 | 0.05 |
| C18 | -0.15 | C18 | -0.18 | HZ2 | 0.05 |
| C19 | -0.15 | C19 | -0.18 | HH1 | 0.33 |
| C20 | -0.18 | C20 | -0.18 | HH2 | 0.33 |
| C21 | -0.15 | C21 | -0.18 | HH3 | 0.33 |
| C22 | -0.15 | C22 | -0.27 |  |  |
| C23 | -0.18 | O | -0.76 |  |  |
| C24 | -0.27 | O24 | -0.76 |  |  |
| H | 0.09 | H | 0.09 |  |  |
| H26 | 0.09 | H26 | 0.09 |  |  |
| H27 | 0.09 | H27 | 0.09 |  |  |
| H28 | 0.09 | H28 | 0.09 |  |  |
| H29 | 0.15 | H29 | 0.09 |  |  |
| H30 | 0.15 | H30 | 0.09 |  |  |
| H31 | 0.09 | H31 | 0.09 |  |  |
| H32 | 0.09 | H32 | 0.09 |  |  |
| H33 | 0.15 | H33 | 0.09 |  |  |
| H34 | 0.15 | H34 | 0.09 |  |  |
| H35 | 0.09 | H35 | 0.09 |  |  |
| H36 | 0.09 | H36 | 0.09 |  |  |
| H37 | 0.15 | H37 | 0.09 |  |  |
| H38 | 0.15 | H38 | 0.09 |  |  |
| H39 | 0.09 | H39 | 0.09 |  |  |
| H40 | 0.09 | H40 | 0.09 |  |  |
| H41 | 0.15 | H41 | 0.09 |  |  |
| H42 | 0.15 | H42 | 0.09 |  |  |
| H43 | 0.09 | H43 | 0.09 |  |  |
| H44 | 0.09 | H44 | 0.09 |  |  |
| H45 | 0.15 | H45 | 0.09 |  |  |
| H46 | 0.15 | H46 | 0.09 |  |  |
| H47 | 0.09 | H47 | 0.09 |  |  |
| H48 | 0.09 | H48 | 0.09 |  |  |
| H49 | 0.15 | H49 | 0.09 |  |  |
| H50 | 0.15 | H50 | 0.09 |  |  |
| H51 | 0.09 | H51 | 0.09 |  |  |
| H52 | 0.09 | H52 | 0.09 |  |  |
| H53 | 0.09 | H53 | 0.09 |  |  |
| H54 | 0.09 | H54 | 0.09 |  |  |
| H55 | 0.09 | H55 | 0.09 |  |  |
|  |  | H56 | 0.09 |  |  |
|  |  | H57 | 0.09 |  |  |
|  |  | H58 | 0.09 |  |  |
|  |  | H59 | 0.09 |  |  |
|  |  | H60 | 0.09 |  |  |
|  |  | H61 | 0.09 |  |  |
|  |  | H62 | 0.09 |  |  |
|  |  | H63 | 0.09 |  |  |
|  |  | H64 | 0.09 |  |  |
|  |  | H65 | 0.09 |  |  |
|  |  | H66 | 0.09 |  |  |
|  |  | H67 | 0.09 |  |  |
